# Assessment of HER2 Protein Overexpression and Gene Amplification in Renal Collecting Duct Carcinoma: Therapeutic Implication

**DOI:** 10.3390/cancers12113345

**Published:** 2020-11-12

**Authors:** Manuela Costantini, Carla Azzurra Amoreo, Liborio Torregrossa, Greta Alì, Enrico Munari, Carmen Jeronimo, Rui Henrique, Sara Petronilho, Umberto Capitanio, Roberta Lucianò, Nazareno Suardi, Maria Teresa Landi, Umberto Anceschi, Aldo Brassetti, Vito Michele Fazio, Michele Gallucci, Giuseppe Simone, Steno Sentinelli, Maria Luana Poeta

**Affiliations:** 1 Department of Urology, IRCCS Regina Elena National Cancer Institute—Rome, via Elio Chianesi 53, 00144 Rome, Italy; manuela.costantini@ifo.gov.it (M.C.); Umberto.anceschi@ifo.gov.it (U.A.); Aldo.brassetti@ifo.gov.it (A.B.); puldet@gmail.com (G.S.); 2 Department of Pathology, IRCCS Regina Elena National Cancer Institute—Rome, via Elio Chianesi 53, 00144 Rome, Italy; carla.azzurra@gmail.com; 3 Department of Surgical, Medical, Molecular Pathology and Critical Area, Anatomic Pathology Section, 56126 Pisa, Italy; libo.torregrossa@gmail.com (L.T.); greta.ali@gmail.com (G.A.); 4 Department of Pathology, Sacro Cuore Don Calabria, 37024 Negrar, Italy; enrico_munari@yahoo.it; 5 Pathology Unit, Department of Molecular and Translational Medicine, University of Brescia, 25123 Brescia, Italy; 6 Cancer Biology and Epigenetics Group, IPO Porto Research Center (GEBC CI-IPOP), Portuguese Oncology Institute of Porto (IPO Porto) & Porto Comprehensive Cancer Center (P.CCC), 4200-072 Porto, Portugal; carmenjeronimo@ipoporto.min-saude.pt (C.J.); henrique@ipoporto.min-saude.pt (R.H.); s.petronilho@gmail.com (S.P.); 7 Department of Pathology and Molecular Immunology, Institute of Biomedical Sciences Abel Salazar (ICBAS), University of Porto, 4050-313 Porto, Portugal; 8 Unit of Urology, Division of Experimental Oncology, Urological Research Institute (URI), IRCCS Ospedale San Raffaele, 20132 Milan, Italy; capitanio.umberto@hsr.it (U.C.); suardi.nazareno@gmail.com (N.S.); 9 Unit of Pathology, IRCCS San Raffaele Scientific Institute, 20132 Milan, Italy; luciano.roberta@hsr.it; 10 Division of Cancer Epidemiology and Genetics, National Cancer Institute, NIH, DHHS, Bethesda, MD 20892, USA; landim@mail.nih.gov; 11 Laboratory of Molecular Medicine and Biotechnology, University Campus Bio-Medico of Rome, 00128 Rome, Italy; fazio@unicampus.it; 12 CNR-Institute of Translational Pharmacology, 00133 Roma, Italy; 13 Department of Urology, University of Rome, La Sapienza, Rome, Viale del Policlinico 155, 00161 Rome, Italy; michele.gallucci@uniroma1.it; 14 Department of Bioscience, Biotechnology and Biopharmaceutics, University of Bari, via Orabona 4, 70126 Bari, Italy

**Keywords:** renal collecting duct carcinoma, HER2, biomarker, IHC, FISH

## Abstract

**Simple Summary:**

Renal collecting duct carcinoma (CDC) is rare, but very aggressive, variant histology of kidney cancers. Besides surgery, the other therapeutic options, such as pharmacological or radiation therapy, have a poor impact on survival. Therefore, there is an urgent need to identify novel targets that can open up new avenues for alternative treatments. From this perspective, the aim of our study was to assess the HER2 protein expression by immunohistochemistry (IHC) and the gene copy number by fluorescence in-situ hybridization (FISH) in a cohort of 26 CDC. According to the 2018 ASCO/CAP guidelines, 2/26 CDC cases (8%) were HER2-positive. The HER2 protein is a well-established target of anti-HER2 mAbs or kinase inhibitors already used for breast and gastric cancer treatment. Thus, this study provides evidence that supports future biomarker-driven clinical trials that could address the lack of therapy, which is still an unmet clinical need for CDC patients.

**Abstract:**

Collecting duct carcinoma (CDC) is rare and aggressive histology of kidney cancers. Although different therapeutic approaches have been tested, the 2-year survival remains very poor. Since CDC exhibits overlapping features with urothelial carcinoma, the analysis of shared molecular alterations could provide new insights into the understanding of this rare disease and also therapeutic options. We collected 26 CDC cases, and we assessed HER2 protein expression by immunohistochemistry (IHC) and gene amplification by fluorescence in-situ hybridization (FISH) according to 2018 ASCO/CAP HER2-testing recommendations. Six out of twenty-six (23%) tumors showed HER2 positive staining. In particular, 3+ score was present in 2/6 cases (33%), 2+ in 3/6 cases (50%) and 1+ in 1/6 cases (17%). The 6 HER2+ tumors were also analyzed by FISH to assess gene copy number. One out of six CDC with IHC 3+ was also HER2 amplified, showing an average HER2 copy number ≥4.0 (10.85) and a HER2/CEP17 ratio ≥ (5.63), while the 5/6 cases were HER2 negative. Based on the 2018 ASCO/CAP guidelines overall, 2/26 CDC cases (8%) were HER2+. The present study provides evidence for testing, in future studies, HER2 to assess its clinical value as a novel target for the treatment of this highly malignant cancer.

## 1. Introduction

Collecting duct carcinoma (CDC) of the kidney, also known as Bellini duct carcinoma, is a rare and aggressive variant histology of renal cell carcinoma (RCC), accounting for 1–2% of all RCC [1]. Early-stage CDC usually undergo radical nephrectomy with curative intent, whereas chemotherapy alone or in combination with radiation therapy in the adjuvant setting is not recommended [2]. Unfortunately, at the time of the diagnosis, about half of the cases have already developed metastasis at lymph nodes, bone, lung, liver, and adrenal glands [3,4]. In these metastatic patients (mCDC), the median overall survival (OS) is about 13 months after diagnosis [5]. Differently from the other and more common renal cancer malignant histologies, such as clear cell renal cell carcinoma, papillary renal cell carcinoma, and chromophobe metastatic, CDC still lacks a standard therapeutic approach [6]. Gemcitabine plus cisplatin chemotherapy is the only recommended therapy for the first-line treatment of mCDC.

Although a phase 2 trial attempted the use of conventional chemotherapy with gemcitabine plus cisplatin in combination with a multitargeted kinase inhibitor as sorafenib, this first-line regimen improved in terms of median PFS of only 8.8 months [7].

More recent studies that used tyrosine kinase inhibitors, such as sunitinib [8], temsirolimus [9], or immune checkpoint inhibitors [10,11,12], mainly represented by case reports or small institution studies, show some improvement of survival.

Overall besides surgical treatment, other therapeutic approaches that include chemotherapy regimens, targeted therapy, immunotherapy [13], and radiation therapy have been proposed for metastatic disease, but survival benefit is still very limited [4,6].

Moreover, the conduction of randomized clinical trials is severely hampered by the low incidence of this RCC histologic variant. CDC arises from the epithelial layer of the distal collecting duct of the kidney, and owing to the common mesonephric origin and the anatomical proximity, CDCs share some clinical, radiologic, morphological, and molecular features with urothelial carcinoma, but it also exhibits various differences [14,15,16]. Based on the similarities, several attempts have been made to test CDCs with different therapeutic agents already used for urothelial carcinoma [17]. Different studies provided evidence that protein overexpression and/or gene amplificationof human epidermal receptor-2 (HER2) occurs in solid tumors, including breast and gastric cancer, enabling the therapeutic use of anti-HER2 mAbs or HER2 kinase inhibitors [18,19] in these tumor types. Likewise, HER2 overexpression and/or gene amplification has also been observed in 0–25% of urothelial carcinoma [20], and it has been considered a target suitable for trastuzumab treatment [21]. HER2 overexpression in CDC has been reported only in a few studies, more specifically in two case-reports [22,23] and one small cohort of 11 cases [24]. Since the lack of effective adjuvant treatment, improved molecular characterization of CDCs, and identifying novel targets that can provide new therapeutic options will be crucial to improve patient outcomes from the perspective of a precision medicine approach. In the present study, we describe different morphological features of 26 CDC cases. Furthermore, we conducted Immunohistochemistry (IHC) and fluorescence in-situ hybridization (FISH) analysis to assess the level of HER2 protein expression and gene amplification according to ASCO/CAP 2018 criteria [25]. This study aims to provide preliminary evidence that can guide future clinical studies to explore HER2-targeting drugs in renal collecting duct carcinoma.

## 2. Results

### 2.1. Clinical and Pathologic Characteristics of CDC Patients

A total of 26 patients diagnosed with CDC in five medical centers were collected and reviewed to confirm the diagnosis. Table 1 summarizes the clinical and pathologic features of the 26 CDC cases included in the study. Among 26 patients, 16 (62%) cases were male and 10 (38%) female. The mean age was 72 years old (range, 40 to 84 years). The average tumor size was 6 cm (range, 2.2 to 10.5 cm). Seven (41%) cases presented distant metastasis at the time of surgery (synchronous lesions), whereas in 10 (59%) patients, the appearance of metastasis was observed after diagnosis (metachronous lesions). Six (35%) patients had metastatic lesions in multiple sites. Tumors were staged according to the 2017 American Joint Committee on Cancer (AJCC) TNM stage classification. Two cases had TNM stage I (8%), 0 stage II (0%), 15 stage III (58%), and 9 stage IV (34%), respectively. Microscopically different architectural patterns have been observed, in particular tubular/solid with confluent solid nests, tubulopapillary, tubulocystic and tubular structures, respectively, present in 12 (46%), 11 (42%), and 2 (8%) and 1 (4%) cases. Additional features that supported the diagnosis of CDC, such as necrosis, desmoplastic stromal reaction, dysplastic changes in adjacent non-neoplastic collecting duct epithelium, intraluminal mucin, presence of Hobnail nuclei, lymphovascular and perineural invasion, pyelonephritis with glomerulosclerosis, sarcomatoid and rhabdoid areas, presence of squamous cells were also observed (Table 1). The inflammatory infiltrates were predominantly represented by lymphocytes and less frequently by the coexistence of lymphocytes and granulocytes (rare eosinophils).

### 2.2. HER2 Immunohistochemical Analysis in CDC

**Figure 1:**
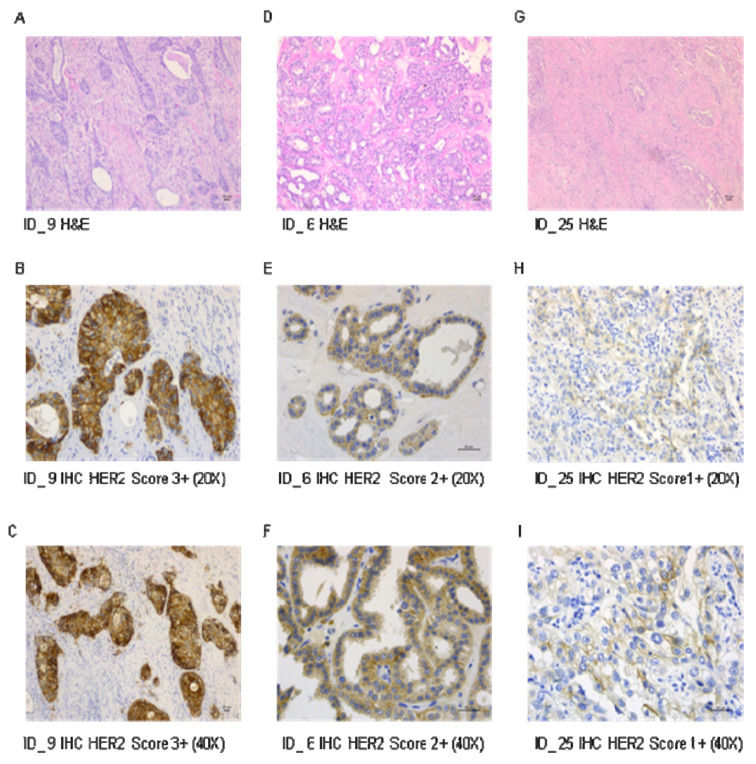
Representative images of H&E staining and IHC analysis in CDC. (**A**) primary tumor withtubulocystic growth pattern. (H&E, ×100). (**B**) Score 3+ HER2 immunohistochemical staining (×20); (**C**) (×40); (**D**) primary tumor with tubulopapillary pattern (H&E, ×100), (**E**) Score 2+ HER2 immunohistochemical staining (×20); (**F**) (×40); (**G**) Lymph node metastasis of CDC (H&E, ×100), (**H**) Score 1+ HER2 immunohistochemical staining (×20) (**I**) (×40).

ASCO/CAP 2018 criteria have been adopted to evaluate the HER2 staining in 26 CDC cases. Six out of twenty-six (23%) patients exhibit cellular membrane positive staining for HER2. In particular, 1/6 cases (17%) had a score of 1+, 3/6 cases (50%) were 2+, and 2/6 cases (33%) were 3+ (Figure 1).

### 2.3. HER2 Fluorescence In-Situ Hybridization Analysis in CDC

**Figure 2:**
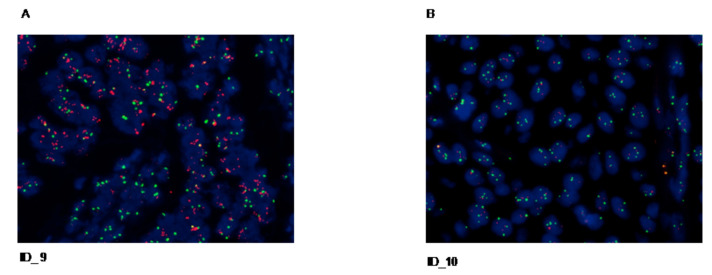
Representative images of fluorescence in-situ hybridization (FISH) analysis of the CDC. Red signals represent HER2 gene copies, while green signals represent chromosome enumeration probe 17 copies (oil fluorescence objective magnification (×60). (**A**) HER2 gene amplification; (**B**) no evidence of HER2 gene amplification.

In this study, all six CDC cases that show positive IHC staining score (1+, 2+, 3+) for HER2 protein expression were tested by FISH to assess HER2 gene copy number (Table 2). FISH results were analyzed by counting the fluorescence signals in at least 20 malignant cells in two different areas of the section at 1000 magnification. For each case, the average HER2 copy number and the ratio of HER2 signals to chromosome 17 centromere (HER2/CEP17) signals were calculated according to the ASCO/CAP 2018. One out of six CDC patients with IHC 3+ was also HER2 FISH positive, showing an average HER2 copy number ≥4.0 (10.85) and a HER2/CEP17 ratio ≥ (5.63) (Figure 2A). The remaining 5/6 cases were regarded as HER2-negative exhibiting HER2/CEP17 ratio <2.0 with an average HER2 copy number <4.0 (Figure 2B). None of the cases analyzed showed HER2-equivocal results (HER2/CEP17 ratio <2.0 with an average HER2 copy number ≥4.0 and <6.0). Overall HER2 test was considered positive when the tumor specimens showed HER2 IHC 3+ or positive HER2 gene amplification by FISH. Considering together IHC and FISH results, we found that 2/26 cases (8%) were HER2 positive (Table 2)

## 3. Discussion

CDC is a rare kidney cancer histotype characterized by an aggressive clinical behavior [1]. Different therapeutic strategies have been tested, including chemotherapies, targeted therapy [7], immunotherapy [10,11,12,13], and radiotherapy, nevertheless, the prognosis still remains very poor [2,6]. Hence, there is an urgent need to provide additional molecular targets and predictive biomarkers, which may be useful for identifying candidate responder patients who may benefit from new treatments. Since CDC exhibits some overlapping features with urothelial carcinoma, different pharmacological agents already tested for urothelial carcinoma [17], have also been attempted in CDC. Because 9–80% of urothelial carcinoma showed HER2 overexpression and about 32% exhibit gene amplification [26], different clinical trials that include anti-HER2 therapies, such as trastuzumab, pertuzumab, lapatinib, and asatinib, used as single agents or in combination with other drugs have been conducted in urothelial carcinoma [17]. To the best of our knowledge, the only three studies that tried to characterize HER2 in CDC include a retrospective study conducted in 11 CDC cases in which HER2 amplification evaluated by competitive PCR, was present in 5 out of 11 cases (45%) and all these patients with HER2 amplified died within one year [24]. Another study carried out HER2 amplification analysis using FISH alone [22] in one patient, whereas another case report performed only IHC analysis showing a focal, faint perceptible membrane staining in less than 10% of the tumor cells [23]. Thus far, no study assessed HER2 expression and amplification status in the same sample cohort of CDC cases. Despite the rarity of the CDC histological subtype, in the present study, we had the chance to collect 26 CDC cases from five different institutions. Since the absence of previous studies that define the HER2 positivity in CDC, we refer to the most recent ASCO/CAP 2018 guidelines [25] to assess the HER2 protein expression by IHC and HER2 gene amplification by FISH in the tumor specimens. Our study revealed that 6 out of 26 patients (23%) exhibit IHC positive staining for HER2 with different scores ranging from 1+ to 3+, in particular, 3/6 cases (50%) were HER2 2+/3+ and 1/6 CDC patient with IHC 3+ was also HER2 FISH positive showing an average HER2 copy number ≥4.0 (10.85) and a HER2/CEP17 ratio ≥ (5.63). According to ASCO/CAP 2018 that considers the HER2 test positive when the tumor specimens showed HER2 IHC 3+ or positive HER2 gene amplification by FISH, we found that 2/26 cases (8%) were HER2 positive. With the exception of one single case report [22], there are no clinical studies that used anti-HER2 compounds in a single or multiple-agent approach in CDC. A large plethora of data indicates that solid tumors with HER2 gene amplification respond to an anti-HER2-targeted therapy [26,27,28,29], with an improvement in clinical outcomes. Based on this principle, our study provides preliminary evidence in support of testing anti-HER2 therapy in CDC. However, our study has some limitations. Indeed, due to the rarity of the disease, and despite the inclusion of five different hospitals in the study, the sample size is still small, leaving unmet needs. Larger studies will indeed be crucial to validate the frequency of HER2 overexpression and/or amplification, to define the clinically relevant threshold of the cut-off score, and to identify the subset of CDC cases that are HER2+ and that could be sensitive to anti-HER2 treatment. Indeed in the present study, the HER2 positivity is based on the breast and gastric cancer HER2 testing criteria, but to consider a pre-specified cut-off value that is routinely used from other tumors to assess the positivity of IHC staining could not identify those CDC cases which may exhibit a response to anti-HER2 therapies. So further studies will need to grade HER2 expression and amplification as values (percentage of the stained tumor cells plus staining intensity for IHC and the average HER2 copy number or HER2/CEP17 ratio for FISH) on a continuous scale to define the cut-offs that can have clinical significance for CDC. In the era of targeted therapies, a stringent evaluation of the gene and/or protein status is needed to significantly improve the drug response. Biomarker-driven studies have revolutionized the clinical trial design shortening the time for drug approval. Indeed FDA has recently approved an increasing number of biomarker-based novel compounds across several histotypes based on early-stage (phase I or II) non-randomized clinical trials [30].

For a rare and very aggressive tumor as CDC, the design of clinical trials and the definition of standard therapies are more challenging than those for major cancers, due to several factors, such as the difficulty of the patient recruitment, the randomization, and the lack of knowledge of molecular alterations.

From this perspective, identifying actionable targets is pivotal for biomarker-driven studies that can provide more effective therapeutic options in CDC patients.

## 4. Materials and Methods

### 4.1. Case Selection and Tissue Specimen Collection

A total number of 26 CDC have been collected, including different institutions, in particular (a) fourteen cases derived from Regina Elena National Cancer Institute of Rome (b) seven cases from Hospital Sacro Cuore Don Calabria Verona, (c) five cases from Pisa University Hospital, (d) four cases from Portuguese Oncology Institute of Porto, and (e) one case from IRCCS Ospedale San Raffaele, Milan. After a first histologic examination on hematoxylin–eosin stained slides carried out in the institution where each case was collected, all tissue specimens underwent a centralized revision by a dedicated uro-pathologist (SS). Only confirmed CDC cases have been further considered for the analysis of HER2 protein expression and gene amplification. For each patient, two representative blocks were selected for immunohistochemistry (IHC) analysis. Tumor tissue specimens were formalin-fixed paraffin-embedded (FFPE), and 3 μm sections were cut from the primary tumor specimens for hematoxylin-eosin staining to inspect the presence of neoplastic cells. The material poorly fixed and/or with low cellularity (<70% neoplastic cells) had been previously excluded. This study was conducted in accordance with the ethical standards of each institutional research committee and the Declaration of Helsinki. The hospital records were used to describe the clinical and pathological features of the cases included in the study (Table 1).

### 4.2. HER2 Immunohistochemical Analysis

For each patient, two paraffin blocks, with at least 70% of neoplastic cells, were selected, and for each block 3, micra tissue sections were cut and used for immunohistochemical (IHC) analysis after transferring them to SuperFrost Plus slides (Menzel-Gläser, Braunschweig, Germany). After deparaffinization, rehydration, and antigen retrieval in citrate buffer (10 mMol, pH 6.1), tissue sections were stained for HER2 (A0485 polyclonal antibody; Dako, Glostrup, Denmark; Dilution 1/200). Immunoreactions were revealed by Bond Polymer Refine Detection on an automated autostainer (Bond™Max, Leica Biosystem, Milan, Italy). Standard processing steps were performed according to the manufacturer’s instructions. As chromogenic substrate Diaminobenzidine was used. The positivity for HER2 was assessed according to recommendations of the American Society of Clinical Oncology/College of American Pathologists 2018 scoring system guideline established for breast cancer, evaluating only membranous staining [25]. The interpretation of the results was also based on the negativity of collecting duct normal tissues. The level of HER2 protein expression was semi-quantitatively evaluated, considering the intensity and the percentage of staining and scored on a scale ranging from 0 to 3+according to ASCO/CAP 2018 guidelines. Scores of 0 and 1+ are categorized as negative, 2+ as equivocal, and 3+ as positive.

### 4.3. HER2 Fluorescence In-Situ Hybridization

All specimens presenting any score 1+ 2+ or 3+ HER2 protein expression were further evaluated by Fluorescence in-situ hybridization (FISH) using two selected blocks. The analysis was performed on 2 to 3 μm thick paraffin sections of tumor tissues using PathVysion Kit (Abbott Molecular Inc., Des Plaines, IL, USA) that is designed for the detection of HER-2/neu gene amplification in formalin-fixed, paraffin-embedded human tissue specimens placed on slides, according to the manufacturer’s instructions. Before hybridization, paraffin sections were deparaffinized in xylene (3 times, 10 min each), dehydrated by two 5 min washes in 100% ethanol, then two 5 min washes in 96% ethanol, and air-dried at room temperature. Tissue sections were then transferred in Vysis Pretreatment Solution (Abbott Molecular Inc., Des Plaines, IL, USA) at 81 °C for 30 min, followed by 3 min washes in purified water, and treated with protease solution (Vysis Protease Buffer IV, Abbott Molecular Inc., Des Plaines, IL, USA) for 10 min at 37 °C to digest proteins. After brief washing in purified water, the slides were sequentially dehydrated in alcohol (70%, 85%, and 100%) and air-dried at room temperature, followed by hybridization with the probe Vysis LSI HER-2/neu Spectrum Orange/Cep 17 Spectrum Green (Abbott Molecular Inc., Des Plaines, IL, USA).

Following hybridization, the unbound probe is removed by a series of washes, and the nuclei are counterstained with DAPI (4,6 diamidino-2-phenylindole), a DNA-specific stain that fluoresces blue. Hybridization of the PathVysion probes is viewed using a fluorescence microscope equipped with appropriate excitation, and the emission filters visualize the intense orange and green fluorescent signals. Enumeration of the LSI HER-2/neu and CEP 17 signals is conducted by microscopic examination of the nucleus, which yields a ratio of the HER-2/neu gene to chromosome 17 copy number. The number of LSI HER-2/neu and CEP 17 signals per nucleus are recorded. Results on the enumeration of 20 interphase nuclei, conducted in two different areas of the section at 1000magnification, from tumor cells per target are reported as the ratio of the total HER-2/neu signals to those of CEP 17. According to ASCO/CAP, 2018 guidelines HER2 positivity by FISH was defined as an average HER2 copy number ≥4 or HER2/CEP17 ratio ≥2.0. The cases showing HER2/CEP17 ratio <2.0 with an average HER2 copy number ≥4.0 and <6.0 were regarded as HER2-equivocal, and the cases showing HER2/CEP17 ratio <2.0 with an average HER2 copy number <4.0 were regarded as HER2-negative. The results of the HER2 test were considered positive when the tumor specimens showed HER2 IHC 3+ or positive HER2 gene amplification by FISH.

## 5. Conclusions

This is the first study to provide a comprehensive evaluation of HER2 in a rare, but very aggressive, histotype (such as CDC), in agreement with the most recent ASCO/CAP 2018 guidelines. These data may pave the way for future biomarker-driven clinical studies to test anti-HER2 strategies in CDC.

## Figures and Tables

**Table 1:** Clinical and pathologic characteristics of collecting duct carcinoma (CDC) patients.

Variable CDC Patients (*N* = 26)	
Gender	*N* = 26
Male	16 (62%)
Female	10 (38%)
Age median (range)	72 (40–84)
Stage	*N* = 26
I	2 (8%)
II	0 (0%)
III	15 (58%)
IV	9 (34%)
T stage	*N* = 26
T1	3 (12%)
T2	0 (0%)
T3	18 (69%)
T4	5 (19%)
N stage	*N* = 26
N0	1 (4%)
N1	9 (34%)
N2	2 (8%)
Nx	14 (54%)
M stage	*N* = 26
M0	6 (23%)
M1	7 (27%)
Mx	13 (50%)
Presence of distant metastasis	*N* = 26
No	9 (35%)
Yes	17 (65%)
Time of metastasis	*N* = 17
Syncronous	7 (41%)
Metachronous	10 (59%)
Site of Metastasis	*N* = 17
Lymph nodes	1 (6%)
Adrenal Gland	2 (12%)
Bone	2 (12%)
Liver	1 (6%)
Lung	5 (29%)
Multiple sites	6 (35%)
Size of the tumor	*N* = 26
median (range cm)	6 (2.2–10.5)
<6	11 (42%)
≥6	15 (58%)
Architectural patterns	*N* = 26
tubular/solid with confluent solid nests	12(46%)
tubulopapillary	11(42%)
tubulocystic	2 (8%)
Tubular	1 (4%)
Necrosis	*N* = 26
Yes	21 (81%)
No	5 (19%)
Desmoplasia	*N* = 26
Yes	24 (92%)
No	2 (8%)
Dysplastic changes in adjacent non-neoplastic collecting ducts	*N* = 26
Yes	16 (61%)
No	10 (39%)
Intraluminal Mucin	*N* = 26
Yes	5 (19%)
No	21 (81%)
Hobinal Cells	*N* = 26
Yes	4 (15%)
No	22 (85%)
Lymphovascular Invasion	*N* = 26
Yes	9 (35%)
No	17 (65%)
Perineural Invasion	*N* = 26
Yes	5 (19%)
No	21 (81%)
Pyelonephritis with Glomerulosclerosis	*N* = 26
Yes	6 (23%)
No	20 (77%)
Sarcomatoid areas	*N* = 26
Yes	10 (39%)
No	16 (61%)
Rhabdoid Areas	*N* = 26
Yes	2 (8%)
No	24 (92%)
Presence of squamous cells	*N* = 26
Yes	9 (35%)
No	17 (65%)
Inflammatory Infiltrate	*N* = 26
Low (1–25%)	11 (42%)
Moderate (25–60%)	13 (50%)
Strong (>60%)	2 (8%)
HER2 IHC Staining	*N* = 26
Negative	20 (77%)
Positive	6 (17%)
HER2 Positive IHC Staining	*N* = 6
1+	1 (17%)
2+	3 (50%)
3+	2 (33%)

**Table 2:** HER2 IHC and FISH results in IHC-HER2+ collecting duct carcinoma (CDC) patients.

ID	HER2_IHC	HER2_FISH	HER2_FISH (Average Copy Number)	HER2_FISH (HER2/CEP1 Ratio)	Overall HER2 Status (Positive/Negative)
6_CDC	2+	Negative	2.2	1	Negative
10_CDC	2+	Negative	3	1.01	Negative
9_CDC	3+	Positive	10.85	5.63	Positive
16_CDC	2+	Negative	2.27	1.02	Negative
11_CDC	3+	Negative	3.3	1.09	Positive
25_CDC	1+	Negative	2.12	1.08	Negative
